# Relapsed/Refractory Follicular Lymphoma: Current Advances and Emerging Perspectives

**DOI:** 10.1111/ejh.14401

**Published:** 2025-02-19

**Authors:** Giulio Caridà, Enrica Antonia Martino, Antonella Bruzzese, Daniele Caracciolo, Caterina Labanca, Francesco Mendicino, Eugenio Lucia, Virginia Olivito, Teresa Rossi, Antonino Neri, Ernesto Vigna, Pierfrancesco Tassone, Pierosandro Tagliaferri, Fortunato Morabito, Massimo Gentile

**Affiliations:** ^1^ Hematology Unit, Department of Onco‐Hematology Cosenza Italy; ^2^ Department of Experimental and Clinical Medicine University of Catanzaro Catanzaro Italy; ^3^ Laboratory of Translational Research Azienda USL‐IRCSS di Reggio Emilia Reggio Emilia Italy; ^4^ Scientific Directorate, Azienda USL‐IRCCS di Reggio Emilia Reggio Emilia Italy; ^5^ Gruppo Amici Dell'Ematologia Foundation‐GrADE Reggio Emilia Italy; ^6^ Department of Pharmacy, Health and Nutritional Science University of Calabria Rende Italy

**Keywords:** bispecific antibodies, CAR‐T, R/R FL, therapy

## Abstract

Follicular lymphoma (FL) is a prevalent indolent non‐Hodgkin lymphoma (NHL) characterized by a relapsing course and eventual refractoriness to therapy. Despite advancements in treatment, FL remains incurable, necessitating ongoing research into novel therapeutic strategies. This review provides a comprehensive overview of current standard treatments for relapsed or refractory (R/R) FL, including chemoimmunotherapy and stem cell transplantation, and delves into emerging therapies such as chimeric antigen receptor (CAR) T‐cell therapy and bispecific antibodies. We discuss the efficacy and safety profiles of these innovative treatments, their integration into existing treatment paradigms, and the potential they hold in altering the natural history of FL. Additionally, we explore the challenges associated with these therapies, including accessibility, cost, and long‐term management of adverse effects. By examining the evolving therapeutic landscape, this review aims to provide insights into future directions for achieving sustained remission and improving the quality of life in patients with R/R FL.

## 1 Introduction

Follicular lymphoma (FL) is the second most common subtype of non‐Hodgkin lymphoma (NHL) in Western countries, with an age‐standardized incidence ranging approximately from 2 to 4 cases per 100 000 person‐years [1]. According to the SEER database, the rate of new cases of FL was 2.5 per 100 000 men and women per year, and the mortality rate was 0.4 per 100 000 men and women per year.

FL is an indolent yet incurable B‐cell malignancy characterized by a heterogeneous clinical trajectory. The majority of patients experience a relapsing/remitting disease course [2], whereas a subset achieves prolonged remission. However, a small proportion of cases undergo histologic transformation into an aggressive lymphoma, most commonly diffuse large B‐cell lymphoma (DLBCL), which is associated with significantly worse prognosis and inferior survival outcomes [3].

Morphologically, FL is characterized by densely packed abnormal follicles lacking polarization and a distinct mantle zone. Histologic grading of FL is based on the proportion of centroblasts (large B cells with prominent nucleoli) to centrocytes (small‐ to medium‐sized cleaved B cells) within the follicles. Grades 1 and 2 of FL are predominantly composed of centrocytes, while Grade 3 is further subclassified into 3A and 3B based on the proportion of centrocytes. Grade 3A retains a significant centrocyte component and is generally managed using treatment approaches similar to the low‐grade paradigm. In contrast, Grade 3B is characterized by a predominance of centroblasts and follows treatment strategies akin to those used for DLBCL [4].

Immunophenotypically, FL is typically CD10+, BCL2+, CD20+, BCL6+, with variable expression of CD23, and is negative for CD43 and CD5 [5].

Therapeutic strategies for FL vary based on the clinical presentation at diagnosis, ranging from active surveillance to intensive immunochemotherapy [6], and some clinical tools can help physicians in treatment decision‐making, including GELF criteria, which assess the need for immediate therapy [7], and FLIPI score, a prognostic index that stratifies patients based on survival outcomes [8].

The first‐line treatment for FL encompasses a range of therapeutic options tailored to the disease stage and patient characteristics. In early‐stage FL or frail patients, radiation therapy is a viable and effective strategy [9]. For those requiring systemic therapy due to symptomatic disease or organ involvement, alkylating agent‐based immunochemotherapy (ICT) represents the standard first‐line approach. Bendamustine in combination with rituximab is commonly utilized [10], followed by rituximab maintenance therapy, as evidenced by the PRIMA trial, which demonstrated significant improvements in both Overall Survival (OS) and Progression‐Free Survival (PFS) with 2 years of maintenance [11]. In advanced‐stage disease, anti‐CD20‐based regimens, including rituximab or obinutuzumab, serve as the cornerstone of therapy, with the GALLIUM trial providing robust clinical support for these approaches [12]. In contrast, high‐grade FL (Grade 3B) typically requires more intensive ICT, such as the R‐CHOP regimen [13], aligning its management with that of DLBCL.

### 1.1 Considerations at Relapse

Despite the efficacy of anti‐CD20‐based regimens in the first‐line setting, the majority of patients with FL eventually experience relapse or disease progression [14]. The management of refractory or relapsed (R/R) FL is influenced by several key factors, including the time to relapse, prior therapies, and patient performance status.

The interval between initial treatment and relapse is a critical determinant in therapeutic decision‐making. Early relapse, particularly within 24 months of frontline therapy (POD24) [15, 16, 17], is associated with a more aggressive disease phenotype and significantly worse survival compared to late relapses. Identifying POD24 patients at an early stage is essential, as they may benefit from more intensive or novel therapeutic strategies. Additionally, the extent of disease burden at relapse plays a crucial role in treatment selection. Patients with bulky disease often necessitate more active therapies to achieve rapid disease control, whereas those with a low‐tumor burden may be candidates for less intensive options, such as immunomodulatory drugs or rituximab monotherapy. A personalized treatment strategy, integrating patient‐ and disease‐specific factors, is essential to optimizing outcomes in the R/R setting.

## 2 Rituximab Re‐Treatment

In patients with FL, rituximab re‐treatment (RR) at disease progression represents an effective alternative to maintenance rituximab (MR), offering sustained disease control with reduced drug exposure. This strategy is particularly relevant for patients with low‐tumor burden FL and was assessed in the RESORT trial (ECOG E4402) [18], which enrolled 408 patients. Of these, 289 achieved a response to induction rituximab therapy and were subsequently randomized to either MR or RR at progression.

The primary endpoint, time to treatment failure (TTF), was similar between the two arms. However, MR was associated with a prolonged time to first cytotoxic therapy, with 95% in the MR cohort remaining free from such treatment at 3 years compared to 84% in the RR group (*p* = 0.03). Importantly, patients in the RR arm received significantly fewer rituximab doses, with a median of four compared to 18 in the MR arm.

Despite the lower cumulative rituximab exposure, patients of the RR cohort maintained durable disease control with high response rates. Both strategies were well tolerated, with no significant differences in health‐related quality of life (HRQOL) and a low incidence of severe toxicities. The results of this trial established the RR approach as a viable alternative to MR, balancing long‐term disease control with a reduced treatment burden.

## 3 Immunochemotherapy

For patients with FL who fail to respond to or experience disease progression within 6 months of treatment with rituximab or rituximab‐containing regimens, the combination of obinutuzumab and bendamustine (O+B) is a recommended therapeutic option. This was demonstrated in the GADOLIN trial [19], a multicenter, open‐label, phase III study that investigated the efficacy of O+B for 6 cycles compared to 6 cycles of bendamustine monotherapy in patients with R/R FL. Patients in the O+B cohort who did not experience disease progression continued obinutuzumab maintenance therapy for up to 2 years. Notably, prior exposure to bendamustine within 2 years of trial enrollment was an exclusion criterion.

PFS, the primary endpoint, was significantly prolonged in the O+B arm, with the median PFS not reached at the time of analysis, compared to 14.9 months with bendamustine (HR 0.55, 95% (CI, 0.40–0.74), *p* = 0.0001). Additionally, the O+B was associated with an extended duration of response and a delayed time to the next treatment.

The GADOLIN trial established the role of ICT in the treatment of R/R FL, particularly in rituximab‐refractory patients, highlighting the potential role of obinutuzumab as an effective therapeutic agent in this setting.

## 4 Immunomodulators

Recent years have seen the emergence of chemo‐free regimens, which are increasingly utilized.

The AUGMENT trial evaluated the addition of lenalidomide to rituximab (R2) in R/R FL. The study enrolled 396 patients, 81% of whom had FL. Notably, the population included both rituximab‐refractory and rituximab‐sensitive patients. This phase III, multicenter, double‐blind, randomized clinical trial demonstrated an improved PFS, the primary endpoint of the study, with a significantly reduced risk of progression (HR 0.46, 95% CI, 0.34–0.62). Although R2 did not achieve statistical significance for OS, the combination significantly improved response rates. Specifically, the rate of Best Responses was 78% in the R2 arm as compared to 53% in the rituximab arm (*p* = 0.0001). Similarly, the rate of complete responses (CR) was 34% in R2 versus 18% in rituximab. R2 was generally well tolerated, with neutropenia and leukopenia as the most frequent Grade ≥ 3 adverse events (AEs) [20]. These results highlight the combination as a beneficial alternative in R/R FL, particularly for patients who can receive rituximab‐based therapy.

Lenalidomide has also been combined with obinutuzumab in the GALEN trial, a phase II, single‐arm study in patients with CD20‐positive R/R FL (WHO Grade 1–3a). The trial enrolled 89 patients across 24 French centers who had received at least one prior Rituximab‐containing regimen. Treatment included lenalidomide (20 mg orally, days 2–22) with obinutuzumab (1000 mg IV) for six 28‐day cycles, followed by a two‐year maintenance. At a median follow‐up of 2.6 years, the overall response rate (ORR) was 79%, with a CR rate of 38%. Two‐year PFS and OS were 65% and 87%, respectively. Common Grade ≥ 3 AEs included neutropenia (43%) and febrile neutropenia (5%) with one treatment‐related death reported [21].

Looking at these results, the efficacy of lenalidomide was comparable when combined with Rituximab or obinutuzumab. To date, the combination of the lenalidomide –rituximab (R2) regimen is approved by both the FDA and EMA and is widely used in clinical practice.

### 4.1 BKT Inhibitors

In the last few years, Bruton Kinase Inhibitors (BTKi) have emerged as promising therapeutic options for R/R lymphomas.

The DAWN trial, a single‐arm, multicenter phase 2 clinical study, investigated the efficacy of Ibrutinib in patients with R/R FL, who had previously received at least one line of therapy, including chemotherapy. The study reported an ORR of 20.9% (95% CI, 13.7%–29.7%) and a CR of 11%, meaning a negative result because it failed to meet its predefined primary endpoint of achieving an ORR with the lower bound of the 95% CI exceeding 18% [22].

Moreover, the addition of Ibrutinib to BR or R‐CHOP in previously treated patients with R/R FL did not show a statistically significant benefit in PFS in the SELENE trial, a study in which Ibrutinib plus ICI was compared against placebo+ICI [23].

Zanubrutinib, another BTKi, was evaluated in combination with obinutuzumab in the ROSEWOOD trial, a phase II, randomized study comparing this regimen to obinutuzumab monotherapy in patients with R/R FL who had received ≥ 2 prior therapies, including anti‐CD20 and an alkylating agent. At a median follow‐up of 20.2 months, the combination therapy demonstrated a significantly higher ORR (69%) compared to obinutuzumab alone (46%; *p* = 0.001) and higher CR rates (39% vs. 19%). Median PFS was 28.0 months for the combination versus 10.4 months for monotherapy (HR, 0.50; 95% CI, 0.33–0.75; *p* < 0.001). The combination therapy was well tolerated, with no unexpected toxicity observed [24]. Based on these results, the FDA and EMA approved the use of zanubrutinib in combination with obinutuzumab in R/R FL.

While the ROSEWOOD trial highlights the good efficacy of BTKi combinations, clinical results with Ibrutinib monotherapy remain modest, with low CR rates and limited DOR. These findings suggest that BTKi monotherapy may be insufficient for achieving sustained remission in R/R FL. Furthermore, no direct comparison between the BTKi combination (zanubrutinib + obinutuzumab) and the lenalidomide‐based regimen (R2) is available. Indeed, the optimal therapeutic choice for these patients remains undefined and should be guided by patient‐specific factors, including prior therapies, tolerability, and clinician expertise.

## 5 CAR‐T

Chimeric antigen receptor (CAR) T‐cell therapy represents a groundbreaking advancement and a paradigm‐shifting modality in the treatment of R/R FL. This approach involves the genetic engineering of autologous T cells to express a CAR targeting specific tumor‐associated antigens, most commonly CD19, and to mediate cytotoxicity. These engineered cells are infused back into the patient to initiate a targeted immune response [25]. CAR T cells are activated upon antigen recognition, proliferate, and mediate direct cytotoxicity against target cells, often triggering robust immune activation. While highly effective, this therapy requires careful monitoring due to associated toxicities such as cytokine release syndrome (CRS) and neurological toxicities [26].

The ZUMA‐5 trial [27] evaluated Axicabtagene Ciloleucel (axi‐cel), a second‐generation anti‐CD19 CAR T‐cell therapy, in 124 patients with R/R FL who had received at least two prior therapies, including anti‐CD20 antibodies with an alkylating agent. Patients underwent leukapheresis, followed by lymphodepleting conditioning with cyclophosphamide and fludarabine, and received a single infusion of axi‐cel. At a median follow‐up of 17.5 months, the therapy demonstrated a remarkable ORR of 92%, with a CR rate of 74%. Responses were observed across subgroups, including patients with high‐risk disease characteristics. However, Grade ≥ 3 AEs were observed in 70% of patients, including CRS (7%) and neurological events (19%). Despite these toxicities, the efficacy outcomes highlight the potential of axi‐cel in this setting.

The ELARA trial investigated Tisagenlecleucel (tisa‐cel), another anti‐CD19 CAR T‐cell therapy, in 97 infused patients with R/R FL who had undergone at least two prior lines of therapy [28]. Patients with Grade 3B FL or already exposed to CAR‐T were excluded. The trial permitted bridging therapy before tisa‐cel infusion. With a median follow‐up of 16.6 months, the trial reported a CR rate of 69.1% and an ORR of 86.2%. Responses were durable, with an estimated 9‐month DOR rate of 86.5% among complete responders. Notably, tisa‐cel exhibited a favorable safety profile, with no Grade ≥ 3 CRS and only 3% of patients experiencing Grade ≥ 3 neurological events, making it a safer alternative for selected patients.

The TRANSCEND trial investigated Lisocabtagene Maraleucel in 256 efficacy‐evaluable patients, including a subgroup with FL [29]. Patients underwent leukapheresis, lymphodepletion chemotherapy, and sequential infusion of CD8+ and CD4+ CAR T cells. The primary endpoint was ORR by independent review. In the FL subgroup, the ORR was 73%, with a CR rate of 53%. The median PFS was not reached at a median follow‐up of 18.8 months. The therapy demonstrated a manageable safety profile, with Grade ≥ 3 CRS in 2% of patients and Grade ≥ 3 neurological events in 10%.

### 5.1 Bispecific Antibodies

The advent of bispecific antibodies (BsAbs) represents a significant advance in the therapeutic landscape of R/R FL [30]. Unlike monoclonal antibodies such as rituximab or obinutuzumab, which target CD20 on B cells, bispecific antibodies simultaneously engage two distinct antigens: CD20 on malignant B cells and CD3 on normal T cells. This dual targeting mechanism forms an immunological bridge that activates T‐cell‐mediated cytotoxicity against CD20‐positive B cells. Additionally, the Fc region of BsAbs can recruit other immune effector cells, potentially triggering antibody‐dependent cellular cytotoxicity (ADCC) [31]. Recent clinical trials highlight the efficacy and safety of several BsAbs in R/R FL, paving the way for novel therapeutic strategies [32].

The ELM‐2 trial, a multicenter, phase II study, evaluated the efficacy and safety of Odronextamab, a CD20 × CD3 BsAb, as intravenous administration in 21‐day cycles, in patients with R/R FL who had received at least two prior therapies [33]. A step‐up dosing regimen in the first cycle mitigated the risk of CRS, a known complication of T‐cell‐engaging therapies [34], starting with a 0.7 mg dose and escalating to 20 mg. Maintenance dosing involved 80 mg weekly for cycles 2–4, followed by 160 mg every 2 weeks or every 4 weeks in patients with CR lasting ≥ 9 months. The treatment continued until disease progression or unacceptable toxicity occurred.

With a median follow‐up of 20.1 months, Odronextamab demonstrated an ORR of 80%, including a CR rate of 73.4%. The median PFS was 20.7 months. Although CRS occurred in 56% of patients, events were predominantly low‐grade (Grades 1–2). The most common Grade 3–4 AE was neutropenia (39%), and 16% of patients discontinued treatment due to AEs.

A phase II multicenter study assessed the efficacy of Mosunetuzumab, another CD20 × CD3 BsAb, in a cohort of 90 patients with histologically R/R FL who were refractory to at least two prior therapies [35]. Using a fixed‐duration treatment regimen, Mosunetuzumab was administered intravenously in 21‐day cycles, with a step‐up dosing schedule during the first cycle to mitigate CRS. Subsequent cycles involved a 30 mg dose on Day 1, up to 8 cycles in patients that achieved a CR; instead, patients that achieved a PR or SD continued the treatment for a maximum of 17 cycles. The enrolled patients had a median of three prior therapies, with 79% refractory to anti‐CD20 antibodies and 53% refractory to any previous anti‐CD20 therapy and an alkylating therapy (double refractory).

The median PFS was 17.9 months, and the median DOR was 22.8 months. CRS was observed in 44% of patients, primarily Grades 1–2, and the overall safety profile was favorable, with only 4% discontinuing treatment due to AEs.

The EPCORE NHL‐1 trial investigated Epcoritamab, a subcutaneous CD3 × CD20 bispecific antibody, in patients with R/R Fl who had received at least two prior lines of therapy [36]. This phase II study included 128 patients treated in the pivotal cohort, with a median of three prior therapies. The treatment schedule involved subcutaneous administration of Epcoritamab in 28‐day cycles: weekly during cycles 1–3, biweekly during cycles 4–9, and monthly thereafter until disease progression or unacceptable toxicity, with step‐up dosing in the first cycle, with doses of 0.16, 0.8, and 48 mg. Prophylactic corticosteroids and hydration were implemented to improve tolerability.

At a median follow‐up of 17.4 months, Epcoritamab achieved an ORR of 82%, including a CR rate of 62.5%. Responses were observed across various subgroups, including those with high‐risk diseases. The median PFS was 18 months, with durable responses maintained in over half of the patients at 18 months. CRS occurred in 66% of patients, primarily Grades 1–2, with Grade 3 CRS reported in only 2% of cases. Other Grade 3–4 AEs included neutropenia (25%) and immune effector cell‐associated neurotoxicity syndrome (6%), which were manageable with no Grade 4–5 cases reported.

Comparative analysis of these trials reveals high efficacy across BsAbs, with ORRs ranging from 80% to 82% and CR rates between 60% and 73.4%.

While Odronextamab demonstrated the highest CR rate, methodological differences between studies, such as patient selection and dosing regimens, may account for these variations.

Median PFS varied slightly across trials: 20.7 months for Odronextamab, 17.9 months for Mosunetuzumab, and 18 months for Epcoritamab. Notably, Epcoritamab's subcutaneous administration provides a logistical advantage over the intravenous delivery required for Odronextamab and Mosunetuzumab, enhancing patient convenience. The dosing regimens also differed: Mosunetuzumab used a fixed‐duration strategy, while Epcoritamab and Odronextamab employed treatment‐to‐progression models, potentially benefiting patients with prolonged responses. However, the fixed‐duration approach of Mosunetuzumab may minimize the risk of cumulative toxicities and probably reduce treatment burdens.

Safety profiles across BsAbs were consistent, with CRS being the most common AE. Epcoritamab recorded the highest CRS incidence (66%) though predominantly lower‐grade events and significant mitigation in the optimized cohort. Severe AEs were infrequent across all studies, though Epcoritamab (19%) and Odronextamab (16%) had higher discontinuation rates compared to Mosunetuzumab (4%).

Emerging evidence suggests that Glofitamab [37] holds promise as a novel therapeutic option, as evaluated in a phase 1, multicenter, open‐label, dose‐escalation, and dose‐expansion clinical trial. The study enrolled 171 patients with various histological subtypes, including 44 patients with R/R FL. The primary objectives were to assess safety, pharmacokinetics, and the maximum tolerated dose of Glofitamab. CRS was the most common AE, occurring in 50.3% of patients, with Grade 3 or 4 CRS in 3.5% of patients. Prior to the initial Glofitamab dose, all patients received 1000 mg of obinutuzumab. Glofitamab was administered in 14‐ or 21‐day cycles, with dose escalation. For patients with FL Grades 1–3A, the ORR was 70.5%, and the CR rate was 47.7%.

BsAbs represent a promising approach in the treatment of R/R FL. High response rates, manageable safety profiles, and novel administration routes underscore their potential. Both BsAbs and CAR‐T are characterized by high rates of ORR and CR and could represent the future of R/R FL therapy. Table 1 provides a comparative summary of BsAbs and CAR‐T therapy outcomes.

**TABLE 1:** Results of clinical trials with BsAbs and CAR‐T for R/R FL.

Therapy type	Trial	Patients (*n*)	ORR (%)	CR rate (%)	Grade ≥ 3 CRS (%)	Grade ≥ 3 neurotoxicity (%)	Median DOR (months)
CAR T‐cell	ZUMA‐5	124	92	74	7	19	NE
CAR T‐cell	ELARA	97	86.2	69.1	0	3	NR
CAR T‐cell	TRANSCEND	73	73	53	2	10	NR
Bispecific antibody	ELM‐2	128	80	73.4	1.7	NA	25.1
Bispecific antibody	Mosunetuzumab	90	80	60	1	NA	22.8
Bispecific antibody	EPCORE NHL‐1	128	82	62.5	0	6	18
Bispecific antibody	Glofitamab	44	70.5	47.7	3.5	0	10.8

Abbreviations: CRS, cytokines release syndrome; DOR, duration of response; NA, not applicable; NE, not extimable; NR, not reached.

### 5.2 PI3K Inhibitors

Phosphatidylinositol 3‐kinase (PI3K) inhibitors, particularly those targeting the δ isoform, have been explored as potential therapeutic agents for R/R FL.

The DELTA trial (Study 101–09), a phase II, open‐label study, assessed Idelalisib, a selective PI3Kδ inhibitor, in 125 patients with relapsed indolent non‐Hodgkin's lymphoma, including 78 with R/R FL refractory to rituximab and alkylating agents. Idelalisib was administered at a dose of 150 mg twice daily until disease progression or unacceptable toxicity. ORR, the primary endpoint, was 57%, with a CR rate of 6%. Despite the promising ORR, the low CR rate raised concerns about the depth of response. Common Grade ≥ 3 toxicities included neutropenia (27%), elevated aminotransferases (13%), diarrhea (13%), and pneumonia (7%), emphasizing the need for careful monitoring during treatment.

When compared to other available therapies, such as R or zanubrutinib‐obinutuzumab, Idelalisib demonstrates lower CR rates and significant toxicity. As a result, its role in the treatment of R/R FL remains uncertain and may be limited to specific patient subgroups or as salvage therapy when other options are unsuitable.

Another PI3K inhibitor, Copanlisib, was investigated in the phase II trial CHRONOS‐1, demonstrating an ORR of 59% and a CR rate of 14%. However, the manufacturer recently announced a collaboration with the FDA to withdraw the drug due to concerns regarding serious AEs.

## 6 Autologous and Allogeneic Transplantation

Although several clinical trials have evaluated auto‐hematopoietic cell transplantation (HCT) and allo‐HCT, after the anti‐CD20 era, the role of stem cell transplantation has become controversial, especially considering the rise of CAR‐T during the last years.

According to the most recent guidelines and consensus [6, 9], auto‐HCT is never an option as consolidation therapy after first‐line treatment but may be considered as consolidation in patients who achieved a CR after salvage treatment, especially in high‐risk patients, such as POD24 lymphomas. Additionally, while auto‐HCT effectively reduces relapse risk, its curative effect is uncertain.

Conversely, allo‐SCT is a potentially curative option for R/R FL due to the graft‐versus‐lymphoma (GVL) effect; however, its use is limited by high non‐relapse mortality (NRM) and the risk of graft‐versus‐host disease (GVHD). Allo‐HCT is typically reserved for patients who have failed multiple lines [38].

### 6.1 EZH2 Inhibitors

Tazemetostat, a first‐in‐class oral Enhancer of zeste homolog 2 (EZH2) inhibitor, has demonstrated promise as a treatment for R/R FL. EZH2 is an epigenetic regulator and is involved in carcinogenesis and cancer development [39]. Tazemetostat efficacy was evaluated in a multicenter, open‐label, phase 1/2 trial involving patients with R/R FL who had received at least two prior systemic therapies [40]. The study stratified patients based on EZH2 mutations (EZH2mut) or wild‐type EZH2 (EZH2WT) mutation status.

The trial population was heavily pretreated, from one to five or more prior lines of therapy. Nearly half (49%) of the EZH2mut group and over half (59%) of the EZH2WT group were refractory to Rituximab‐containing regimens. Additionally, some patients had prior exposure to PI3K inhibitors or immunomodulatory agents. Eligible participants received 800 mg of tazemetostat orally twice daily in continuous 28‐day cycles until disease progression, unacceptable toxicity, or a maximum of 2 years of treatment. The primary endpoint was ORR, with secondary endpoints including DOR and PFS.

The results of the trial revealed significant differences in efficacy between the EZH2mut and EZH2WT cohorts. Among patients with EZH2 mutations (*n* = 45), the ORR was 69% (95% CI 53–82) with a CR rate of 13%. In contrast, patients with EZH2WT disease (*n* = 54) achieved an ORR of 35% (95% CI 23–49) with a CR rate of 4%. These results highlight the enhanced sensitivity of EZH2mut FL to EZH2 inhibition, which aligns with the underlying biology of the mutation driving oncogenesis.

Interestingly, while the ORR was higher in the EZH2mut cohort, the median DOR and PFS were relatively comparable between the two groups. Patients with EZH2mut disease experienced a median DOR of 10.9 months (95% CI 7.2–NE) and a median PFS of 13.8 months (95% CI 10.7–22.0). Similarly, those with EZH2WT disease had a median DOR of 13.0 months (95% CI 5.6–NE) and a median PFS of 11.1 months (95% CI 3.7–14.6). These findings may suggest that while EZH2 mutations enhance initial responsiveness, tazemetostat probably provides durable benefits across both molecular subtypes of FL.

The favorable safety profile of tazemetostat further supports its utility; indeed, no Grade 4 AEs were reported, and all Grade 3 AEs have not had an incidence greater than 1%. As an oral agent, it offers convenience and may be particularly beneficial for patients who are unable to tolerate intensive systemic therapies.

## 7 Expert Opinion

The therapeutic landscape for R/R FL has evolved significantly with the emergence of novel agents and treatment paradigms, including real‐world data that validate and expand upon clinical trial findings. In this respect, the LEO CReWE real‐world cohort of Mosunetuzumab‐treated patients demonstrated a 12‐month PFS rate of 58%, with ORR and CR rates of 73% and 53%, respectively [41].

Additionally, real‐world registries confirm the efficacy and safety of therapies like axi‐cel and tisa‐cel, demonstrating durable remissions even in high‐risk subgroups [42, 43]. However, logistical challenges, including manufacturing timelines and costs, remain significant barriers to widespread implementation. The role of CAR T‐cell therapy in earlier treatment lines is a topic of active investigation and could further optimize outcomes in this patient population. The integration of these insights is crucial for bridging the gap between controlled trial environments and diverse, real‐world patient populations. When considering sequencing, CAR‐T therapy may be prioritized for younger, fit patients with high‐risk or aggressive diseases who are likely to benefit from its curative potential. Conversely, BsAbs may be more appropriate for older or less fit patients, those with lower tumor burdens, or those who have relapsed after CAR‐T therapy.

Real‐world evidence supports the efficacy of the R2 regimen in routine clinical practice, complementing data from the Phase III AUGMENT trial, which demonstrated a median PFS of 39.4 months. A real‐world study reported an ORR of 82% and a median PFS of 22 months, confirming its utility across diverse clinical scenarios, including rituximab refractoriness and prior malignancies.

Combination therapies and novel agents also hold promise for reshaping treatment paradigms. The obinutuzumab/lenalidomide regimen (GALEN) and emerging options such as tazemetostat and BsAbs have shown encouraging efficacy and manageable safety profiles in real‐world settings.

In parallel, the role of autologous and allogeneic HCT is evolving. While auto‐HCT remains a consideration for select patients achieving CRs after salvage therapy, particularly those with POD24 lymphomas, its utility has declined in the rituximab era due to concerns about long‐term toxicities and lack of curative potential. Conversely, allo‐HCT, with its GVL effect, offers a potentially curative option but is reserved for patients with chemoresistant disease or relapse after CAR T‐cell therapy, owing to significant risks such as GVHD.

Future directions in FL management must focus on integrating real‐world data with clinical trial results to validate chemo‐free approaches and optimize personalized treatment strategies. The combination of novel agents, innovative therapies, and RWD‐driven insights offers new hope for addressing high‐risk features, such as POD24, and achieving durable remissions or potentially curative outcomes. Multidisciplinary collaboration, patient‐centered care, and ongoing innovation are pivotal to advancing the field and improving the quality of life for patients with R/R FL.

### 7.1 New Perspectives

The present scenario may change very soon thanks to intense research in the field. Ongoing trials are investigating new molecules or new combinations, and some appear to be promising. Indeed, starting from the promising results of new molecules previously described, such as BTK inhibitors, PI3K inhibitors, EZH inhibitors, and BA, some ongoing trials are evaluating combinations of them with CR as a primary endpoint. Emerging data indicate that CD20 BsAbs are being tested in earlier lines of therapy (OLYMPIA‐2, OLYMPIA‐5), with the potential to shift treatment paradigms by reducing the reliance on conventional chemoimmunotherapy or delaying the need for CAR‐T cell therapy.

New promising and outstanding results come from the inMIND trial [36], recently presented at ASH 2024. The inMIND trial is a phase 3, randomized, double‐blind trial that evaluated the addition of Tafasitamab+R2 versus R2 in R/R FL patients. The trial enrolled 548 patients who had received at least one prior systemic therapy.

PFS, the primary endpoint, showed a significant benefit for Tafasitamab+R2, with a median PFS of 22.4 months compared to 13.9 months for R2 (HR 0.43, 95% CI, 0.32–0.58, *p* < 0.0001). Secondary outcomes, including ORR (83.5% vs. 72.4%) and complete metabolic response (49.4% vs. 39.8%), also favored the Tafasitamab+R2 arm.

The safety profile of Tafasitamab+R2 was manageable, with similar rates of Grade 3 or higher AEs compared to R2 alone. Neutropenia and pneumonia were among the most common toxicities.

The trial supports Tafasitamab+R2 as a potential new standard for patients with R/R FL, offering significant improvements in disease control and durability while maintaining a tolerable safety profile suitable for widespread clinical use.

**FIGURE 1:**
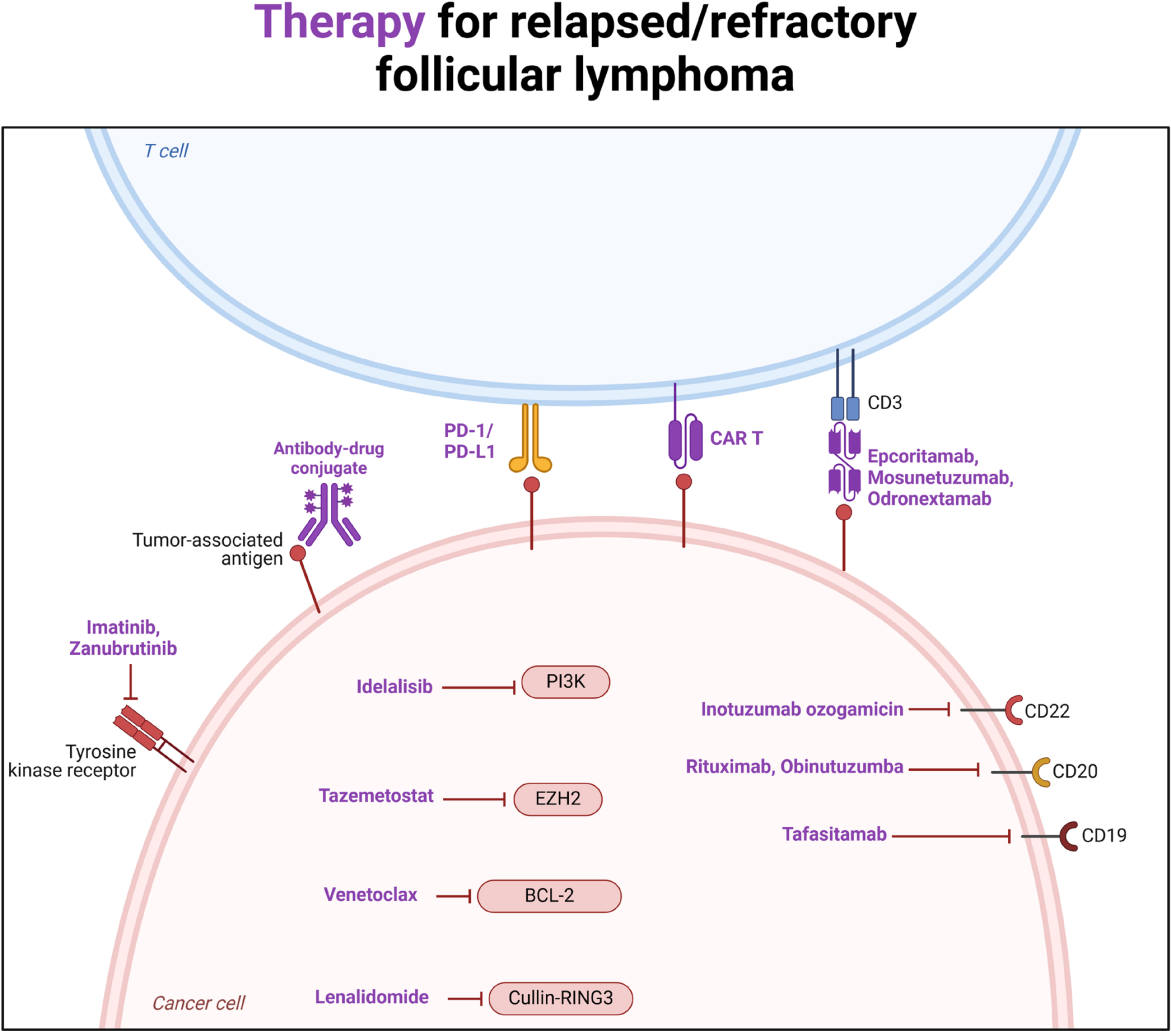
Mechanism of action of new drugs for R/R FL.

In Table 2, ongoing trials involving new molecules or new combinations of drugs are briefly reported, whereas the mechanisms of described drugs are summarized in Figure 1.

**TABLE 2:** Ongoing trials involving new molecules or new combinations of drugs for R/R FL.

NCT code	Phase	Experimental therapy	Endpoint
NCT04224493	III	Tazemetostat in combination with lenalidomide plus rituximab	PFS, safety
NCT02956382	I/II	Ibrutinib plus venetoclax	Recommended dose, ORR
NCT04587687	II	Brentuximab vedotin plus bendamustine	CR
NCT06492837	II	Mosunetuzumab plus zanubrutinib	CR
NCT06368167	II	SHR2554	ORR
NCT03600441	II	Abexinostat	ORR
NCT04998669	II	Loncastuximab tesirine plus rituximab	CR
NCT03269669	II	Obinutuzumab plus lenalidomide or umbrelasib or CHOP	CR
NCT02390869	III	Rituximab plus lenalidomide versus rituximab alone	PFS
NCT06575686	II	Epcoritamab plus tazemetostat	Safety, CR
NCT06453044	II	Mosunetuzumab plus polatuzumab vedotin	Safety, CR
NCT04712097	III	Mosunetuzumab plus lenalidomide versus rituximab plus lenalidomide	PFS
NCT06563596	II	Epcoritamab plus zanubrutinib and rituximab	CR
NCT06149286	III	Odronextamab plus lenalidomide versus rituximab plus lenalidomide	Safety, PFS
NCT06097364	III	Odronextamab plus CT (O‐CHOP) versus R‐CHOP	Safety, CR
NCT03401853	II	Pembrolizumab plus rituximab or obinutuzumab	ORR
NCT04680052	III	Tafasitamab plus lenalidomide and rituximab versus placebo plus lenalidomide and tituximab	PFS
NCT02568553	I	Lenalidomide plus blinatumomab	Safety
NCT05683171	I/II	Valemetostat plus rituximab and lenalidomide	Safety
NCT03015896	I/II	Nivolumab plus lenalidomide	Safety, maximum dose
NCT05618366	I	Tazemetostat and venetoclax	Maximum dose
NCT02992522	I	Obinutuzumab, venetoclax, and lenalidomide	Maximum dose
NCT06526793	II	AZD0486	ORR
NCT03150329	I	Pembrolizumab plus vorinostat	Safety
NCT04578600	I	CC‐486, lenalidomide, and obinutuzumab	Safety
NCT04305444	II	DTRM‐555	CR, PR
NCT05453396	II	Loncastuximab tesirine	ORR
NCT04775745	I	LP‐168	Maximus dose
NCT05008055	II	Capivasertib	ORR
NCT03547115	I	Voruciclib alone or plus venetoclax	Maximus dose, safety
NCT05950165	I	CHO‐H01 alone or plus lenalidomide	Safety, ORR
NCT00717925	I	Inotuzumab ozogamicin	Safety, ORR

Abbreviations: CR, complete remission; ORR, overall response rate; PFS, progression‐free survival; PR, partial remission.

## 8 Conclusions

The therapeutic landscape for R/R FL has undergone transformative advancements in recent years, fueled by the development of innovative immunotherapies and targeted treatments. CAR T‐cell therapies and BsAbs have demonstrated unprecedented clinical efficacy, with high CR rates and prolonged DOR across various patient populations. Despite these advances, R/R FL remains an incurable disease for most patients, emphasizing the need for continued innovation.

The emergence of novel therapeutic targets, such as BTK, PI3K, and EZH2, as well as optimized combination regimens, holds great promise for improving outcomes. Future research must focus on integrating these advancements into standard treatment paradigms and identifying the optimal sequencing of therapies to maximize efficacy while minimizing toxicity.

## Author Contributions

All authors contributed to the manuscript and were involved in revisions and proofreading. All authors approved the submitted version.

## Conflicts of Interest

The authors declare no conflicts of interest.

## Data Availability

The authors have nothing to report.
